# EMC6 regulates acinar apoptosis via APAF1 in acute and chronic pancreatitis

**DOI:** 10.1038/s41419-020-03177-3

**Published:** 2020-11-11

**Authors:** Jie-hui Tan, Rong-chang Cao, Lei Zhou, Zhi-tao Zhou, Huo-ji Chen, Jia Xu, Xue-mei Chen, Yang-chen Jin, Jia-yu Lin, Zhao-chang Qi, Jun-ling Zeng, Shu-ji Li, Min Luo, Guo-dong Hu, Jin Jin, Guo-wei Zhang

**Affiliations:** 1 Division of Hepatobiliopancreatic Surgery, Department of General Surgery, Nanfang Hospital, Southern Medical University, Guangzhou, China; 2 Department of the Electronic Microscope Room, Central Laboratory, Southern Medical University, Guangzhou, China; 3 School of Traditional Chinese Medicine, Southern Medical University, Guangzhou, China; 4 Department of Pathophysiology, Southern Medical University, Guangzhou, China; 5 Department of Occupational Health and Medicine, Guangdong Provincial Key Laboratory of Tropical Disease Research, School of Public Health, Southern Medical University, Guangzhou, China; 6 The First Clinical Medical College, Southern Medical University, Guangzhou, China; 7 Laboratory Animal Research Center of Nanfang Hospital, Southern Medical University, Guangzhou, China; 8 Guangdong-Hong Kong-Macao Greater Bay Area Center for Brain Science and Brain-Inspired Intelligence, Southern Medical University, Guangzhou, China; 9 Department of Laboratory Medicine, Nanfang Hospital, Southern Medical University, Guangzhou, China; 10 Department of Respiratory and Crit Care Medicine, Nanfang Hospital, Southern Medical University, Guangzhou, China; 11 Department of Gynaecology and Obstetrics, Nanfang Hospital, Southern Medical University, Guangzhou, China

**Keywords:** Molecular biology, Pancreatitis

## Abstract

Treatment of acute pancreatitis (AP) and chronic pancreatitis (CP) remains problematic due to a lack of knowledge about disease-specific regulatory targets and mechanisms. The purpose of this study was to screen proteins related to endoplasmic reticulum (ER) stress and apoptosis pathways that may play a role in pancreatitis. Human pancreatic tissues including AP, CP, and healthy volunteers were collected during surgery. Humanized *PRSS1* (protease serine 1) transgenic (*PRSS1*^*Tg*^) mice were constructed and treated with caerulein to mimic the development of human AP and CP. Potential regulatory proteins in pancreatitis were identified by proteomic screen using pancreatic tissues of *PRSS1*^*Tg*^ AP mice. Adenoviral shRNA-mediated knockdown of identified proteins, followed by functional assays was performed to validate their roles. Functional analyses included transmission electron microscopy for ultrastructural analysis; qRT-PCR, western blotting, co-immunoprecipitation, immunohistochemistry, and immunofluorescence for assessment of gene or protein expression, and TUNEL assays for assessment of acinar cell apoptosis. Humanized *PRSS1*^*Tg*^ mice could mimic the development of human pancreatic inflammatory diseases. EMC6 and APAF1 were identified as potential regulatory molecules in AP and CP models by proteomic analysis. Both EMC6 and APAF1 regulated apoptosis and inflammatory injury in pancreatic inflammatory diseases. Moreover, APAF1 was regulated by EMC6, induced apoptosis to injure acinar cells and promoted inflammation. In the progression of pancreatitis, EMC6 was activated and then upregulated APAF1 to induce acinar cell apoptosis and inflammatory injury. These findings suggest that EMC6 may be a new therapeutic target for the treatment of pancreatic inflammatory diseases.

## Introduction

Acute pancreatitis (AP) is characterized by persistent inflammation in the pancreas. Local and systemic inflammatory response syndrome resulting from AP can impair the functions of other organs and may progress to necrosis of the pancreas or surrounding fatty tissue^1^. AP patients who have persistent organ failure with infected pancreatic necrosis have the highest risk of death^2,3^. Currently, there is no curative therapy for AP. Fluid resuscitation and pain control remain the key elements for AP treatment^3,4^. Moreover, recurrent AP, as seen in hereditary pancreatitis, is prone to develop into chronic pancreatitis (CP), which is commonly regarded as having a strong association with pancreatic cancer^5^. Until now, specific drug targets for AP and CP have not yet been identified due to lack of clarity in the pathological mechanism of pancreatitis^6,7^.

Recent studies from our laboratory and other groups have shown that, endoplasmic reticulum (ER) stress is the main pathological mechanism of AP^8–10^. ER stress responses are normal homeostatic mechanisms required for the physiological function of the pancreas of synthesizing large amounts of digestive enzymes, which occurs in the abundant ER of pancreatic acinar cells^11^. However, severe ER stress, following an accumulation of misfolded proteins during pancreatitis, is a causative trigger of acinar cell apoptosis^11,12^, and may promote inflammatory reactions in the surrounding tissues, accelerate disease progression or create secondary damage^9,11,13^. Using the humanized *PRSS1*^*Tg*^ mouse model of CP, we previously showed that ER stress-mediated dysregulation of acinar cell apoptosis induces acinar injury and promotes the development of CP^14^. Thus, ER stress and apoptosis play important roles in pancreatic inflammatory diseases.

A lack of suitable animal models for mechanistic research and drug screening has further limited progress in AP research. In view of this, we established humanized *PRSS1*^*Tg*^ mice that could mimic the progression of human AP and CP. Using these mice to screen for ER stress-associated and apoptosis-related proteins involved in pancreatitis development, we identified ER membrane protein complex subunit 6 (EMC6) and apoptotic peptidase activating factor 1 (APAF1) as potential regulatory proteins in pancreatic inflammatory disease. The expression levels of EMC6 and APAF1 were markedly increased during pancreatitis progression and that APAF1 was positively regulated by EMC6. These results suggest, for the first time, that modulating the expression of EMC6 and/or APAF1 could prevent the progression of pancreatic inflammatory diseases, and provide the basis for the development of new drug targets to treat pancreatitis.

## Materials and methods

### Human pancreatic tissue and animal study

Human pancreatic tissues were collected from six AP patients (mean age = 48.33 ± 5.05), 13 CP patients (mean age = 46.92 ± 17.60), and 12 patients with benign pancreatic tumors or peritumoural normal pancreatic tissues (mean age = 46.83 ± 12.91) as controls (Supplemental Table S1). All patients were administrated to the Division of Hepatobiliopancreatic Surgery, Department of General Surgery, Nanfang Hospital, Southern Medical University, between 2017 and 2019. Written consent was obtained from each patient before the study, which were approved by the Ethics Committee of the Southern Medical University.

Healthy C57BL/6 mice aged 5–6 weeks were used in this study. Mice were housed on standard experimental cages at 24 ± 2 °C under a 12-h light/12-h dark cycle, and were supplied with standard laboratory animal chow and free access to water. The experimental operations were carried out in strict accordance with the Guide for the Care and Use of Laboratory Animals of the National Institutes of Health and were approved by the Institutional Animal Care and Use Committee of Southern Medical University.

### PRSS1^Tg^ mice, adenoviral shRNA for EMC6 and APAF1 knockdown

Humanized *PRSS1*^*Tg*^ (GenBank Accession Number: NM_002769.4) mice^10,14^ were constructed. Adenovirus vectors harboring shRNA for *EMC6* (NCBI Reference Sequence: NM_001168470.1) or *APAF1* (NCBI Reference Sequence: NM_001042558.1) inhibition, or scrambled shRNA, were also constructed. Please see the Supplemental Methods for more details.

### AP and CP induction in PRSS1^Tg^ mice

To establish an AP animal model, *PRSS1*^*Tg*^ mice were intraperitoneally injected with 15 μg/mL caerulein dissolved in phosphate-buffered saline (PBS) at 50 μg/kg every hour for a total of eight injections and sacrificed 24 h later. As described previously^10^, the CP mouse model was induced by injecting *PRSS1*^*Tg*^ mice with caerulein (15 μg/mL) at a dose of 50 μg/kg, one injection per hour for 8 h, twice per week for four consecutive weeks. Wild-type (C57BL/6J) mice undergoing the same treatment schedule were used as the control group.

### Proteomics

For proteomic analyses, tandem mass spectrometry (MS/MS) data were collected for peptide identification through automated data-dependent acquisition (DDA). Using DDA, we used mass information on intact peptides in a full-scan mass spectrum (MS1) to determine which subset of mass signals should be targeted for the further acquisition of fragmentation (MS/MS) spectra to identify peptide sequences.

For protein extraction, quality control and proteolysis, DDA fractions and data independent acquisition (DIA) sample analysis were both performed on a Q Exactive HF X mass spectrometer (Thermo Fisher Scientific) coupled with an Ultimate 3000 RSLC nano system (Thermo Fisher Scientific). DDA data were identified through the Andromeda search engine within MaxQuant, and Spectronaut™ and the identification results were used for spectral library construction. For large-scale DIA data, Spectronaut™ using the constructed spectral library information to complete deconvolution and extraction, and using the mProphet algorithm to complete analytical quality control, was performed to obtain a large number of reliable quantitative results. This pipeline also performed Gene Ontology (GO), clusters of orthologous groups (COG), pathway functional annotation and time series analyses. To select the proteins related to the development of AP, differentially expressed proteins (DEPs) were identified by meeting either of the following criteria: (i) with a significant fold change of >1.5 in the caerulein-treated group compared with the saline-treated group; or (ii) only present in the caerulein-treated group but not in the saline-treated group. Moreover, functional enrichment, protein–protein interaction (PPI) and subcellular localization analyses of the DEPs were performed.

### Transmission electron microscopy (TEM)

For ultrastructural analysis, human pancreatic tissue (1 mm^3^ in size) and mouse pancreatic tissue (1 mm^3^ in size) were fixed with 2.5% glutaral at room temperature for 1 h and then at 4°C overnight. After fixation, the samples were rinsed three times in PBS for 10 min each time. The tissues were then processed for TEM following standard procedures^15^. Finally, ultrathin sections were examined with an electron microscope (Hitachi H-7500, Japan) operated at 60 kV.

### Co-immunoprecipitation

To identify physiological interactions between EMC6 and APAF1 in AP acinar cells, immunoprecipitation was carried out according to the manufacturer’s instructions. Briefly, tissue homogenate was incubated with immunoprecipitation lysate (Servicebio) overnight at 4°C, followed by centrifuging for 10 min at 14,000 × *g*. After the supernatant was collected and the protein concentration was measured by BCA protein quantitative detection kit (G2026; Servicebio, China), a small amount of tissue lysate was collected for input experiments. The tissue lysate was added with 1.0 μg of rabbit IgG (Servicebio) and 20 μl of A /G beads (Millipore) and incubated at 4 °C for 1 h. After centrifugation, the supernatant was incubated with respective primary antibodies against EMC6 (Omnimabs; 2 μg), APAF1 (Proteintech; 2 μg) overnight at 4 °C and rabbit IgG (Servicebio) served as negative control. Similarly, the protein complex was incubated with 80 μl A/G beads (Millipore) at 4 °C for 2 h, then, centrifuged at 1000 × *g* for 5 min at 4 °C to collect the immunoprecipitation complex. After washing several times, boiled in boiling water for 10 min and centrifuged at 4 °C at 1000 × *g* for 5 min to collect the supernatant. Finally, Supernatant was analyzed by immunoblotting with EMC6 (Omnimabs; diluted 1:1000) and APAF1 (Proteintech; diluted 1:1000) antibody.

### Immunohistochemistry and immunofluorescence assay

H&E staining and immunohistochemistry assay of EMC6, APAF1, collagen I, MPO, Caspase-3, and PARP were performed. Immunofluorescence assay of EMC6, APAF1, and α-SMA was also performed. Please see the Supplemental Methods for more details.

### Western blotting and qRT-PCR

Using the Tissue Total Protein Extraction Kit to extract total protein from the mice pancreatic tissues following the manufacturer’s instructions. Primary antibodies used in this study include anti-EMC6, anti-APAF1, and anti-β-actin. The mRNA expression levels of EMC6 and APAF1 in this study were determined by Quantitative RT-PCR. The sequences of the primers used are listed in Supplemental Table S2. Please see the Supplemental Methods for more details.

### Transferase-mediated d-UTP nick-end-labeling (TUNEL) assay

A TdT Frag DNA Fragmentation Imaging kit was used to determine apoptosis according to the manufacturer’s instructions. Please see the Supplemental Methods for more details.

### Enzyme-linked immunosorbent assay (ELISA)

Supernatants were collected and stored at −80 °C after the removal of pancreatic tissues by centrifugation. The protein concentrations of IL-1β, IL-6, and TNF-α in the pancreatic tissues were detected using specific ELISA kits (ROCHE COBAS 8000 E602, Switzerland) according to the manufacturer’s instructions.

### Statistical analysis

Statistical analysis was carried out using GraphPad Prism 8.0 (San Diego, CA, USA) and SPSS 24.0 software (Chicago, IL, USA). All experiments were performed in triplicate with the mean and standard error of the mean (SEM) reported. Significant differences between two groups were analyzed by Student’s *t*-test, and one-way analysis of variance was performed to investigate the differences between more than two groups (ns = no significance; *P < 0.05; **P < 0.01; ***P < 0.001).

## Results

### Human AP and CP tissues exhibit ER stress, mitochondrial damage and apoptosis

**Fig. 1:**
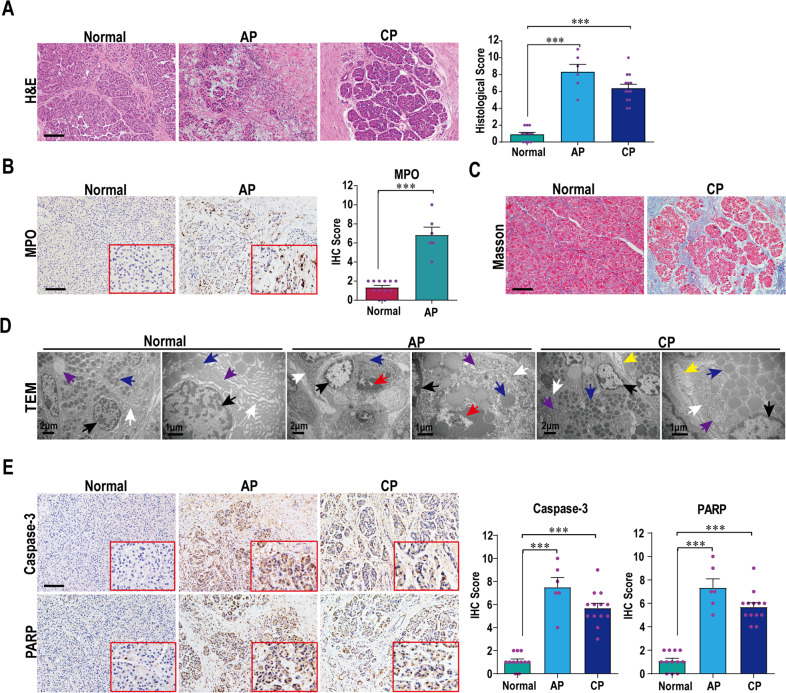
Endoplasmic reticulum (ER) and mitochondria destruction in human acute pancreatitis (AP) and chronic pancreatitis (CP) tissues accompanying with elevated apoptosis. **A** Histological evaluation of human healthy, AP and CP pancreatic tissues by haematoxylin and eosin (H&E) staining and histological score. Pancreas sections from AP patients were stained for myeloperoxidase (MPO) (**B**) while CP tissues were stained for Masson’s trichrome (**C**). **D** The microstructure of human healthy, AP and CP pancreatic tissues were observed by transmission electron microscopy (TEM); black arrows (↑): cell nucleus; white arrows (↑): endoplasmic reticulum; blue arrows (↑): zymogen granule; purple arrows (↑): mitochondria; red arrows (↑): apoptotic body; yellow arrows (↑): mesenchyme. **E** The expression of apoptosis related proteins Caspase-3 and PARP (poly ADP-ribose polymerase) in human pancreatic tissues (including normal, AP and CP) were analyzed. AP acute pancreatitis, CP chronic pancreatitis. Data represents the mean ± SD; *P ≤ 0.05, **P ≤ 0.01. Scale bars = 100 μm.

To figure out whether ER stress and apoptosis existed in human pancreatic inflammatory diseases, we collected pancreatic tissues from healthy volunteers, AP and CP patients for histological analysis (Supplemental Table S1). Compared with healthy tissues, pancreatic acinar cell deformation and inflammatory cell infiltration was significantly increased in AP and CP tissues, which was supported by the increased histological scores (Fig. 1A). Compared with healthy tissues, myeloperoxidase (MPO) expression (Fig. 1B) was increased in AP tissues, while CP tissues exhibited increased collagen expression in the periacinar areas, vacuolization of acinar cells, and substantial pancreatic impairment (Fig. 1C). Further analysis of the microstructure of pancreatic acinar cells by TEM revealed significant pathological alterations, including karyopyknosis, apoptosis, mitochondrial vacuolization, dilatation, and disruption of the ER in AP and CP tissues, which were not observed in healthy tissues (Fig. 1D). Since we previously observed increased pancreatic acinar cell apoptosis in a mouse model of pancreatitis^14^, we examined the expression of apoptotic markers, Caspase-3 and PARP (poly ADP-ribose polymerase) in human AP and CP tissues. The expression of both markers was significantly increased (Fig. 1E). These results indicate ER and mitochondrial damage along with elevated apoptosis in human AP and CP tissues, which strongly suggests a role for ER stress and apoptosis in the development of human pancreatic inflammatory diseases.

### PRSS1^Tg^ mice mimic the development of human AP and CP

**Fig. 2:**
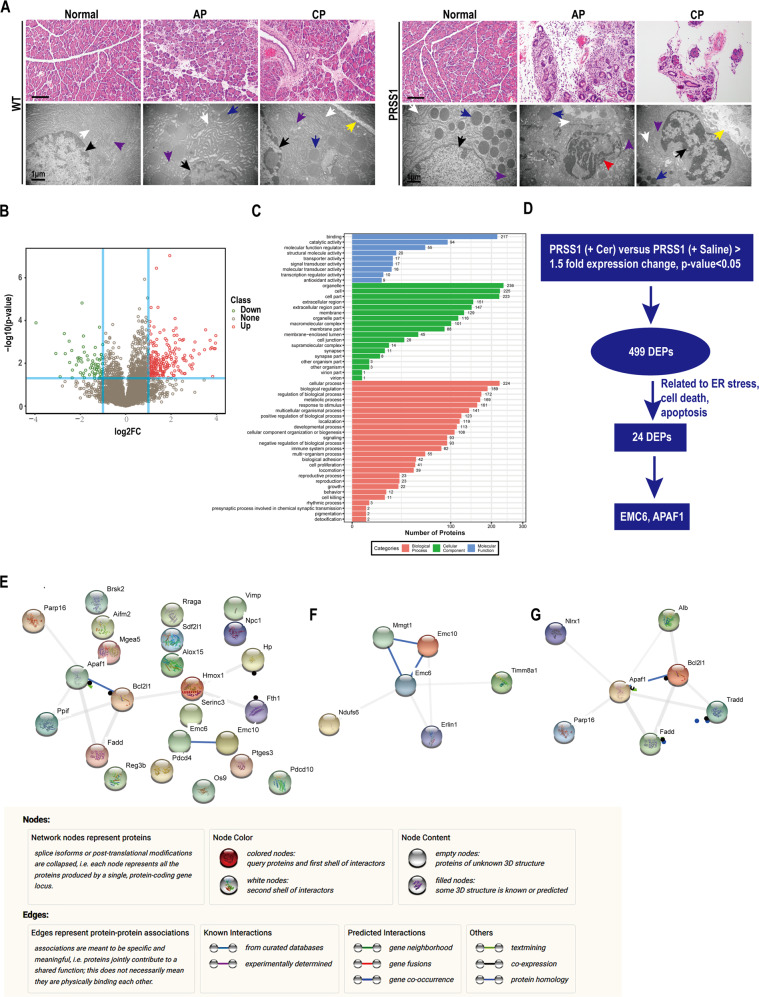
Proteomic analysis identifies potential regulatory proteins related to ER stress and apoptosis during pancreatitis progression. **A** Histological and microstructural evaluation of pancreatic tissues collected from *PRSS1*^*Tg*^ AP, CP mice model and wild-type (WT) mice by H&E staining and TEM, respectively; black arrows (↑): cell nucleus; white arrows (↑): endoplasmic reticulum; blue arrows (↑): zymogen granule; purple arrows (↑): mitochondria; red arrows (↑): apoptotic body; yellow arrows (↑): mesenchyme. **B** Volcano plot, the *X*-axis of the graph was the protein fold change (log 2), and the *Y*-axis was the corresponding −log10 (*P*-value); the red dot indicates significantly upregulated proteins, the green dot indicated significantly downregulated proteins, and the gray dot indicated proteins without significant change. **C** Gene Ontology (GO) function annotation, the GO classification map showed the distribution of the entries involved in the biological process, cellular component, and molecular function. **D** Schematic of proteins related to ER stress, cell death, and apoptosis analysis. **E** The protein–protein interaction (PPI) networks of 24 DEPs constructed in the STRING database. EMC6-related (**F**) and APAF1-related (**G**) PPI networks constructed in the STRING database.

To further determine whether *PRSS1*^*Tg*^ mice could mimic human AP and CP, these mice were administered different treatment schedules of caerulein to induce AP or CP. In comparison to caerulein-treated wild-type (WT) mice, AP or CP phenotype were more obvious in caerulein-treated *PRSS1*^*Tg*^ mice, as reflected by greatly increased histologic and microscopic changes. There was a marked increase in inflammatory cell infiltration in the pancreatitis tissue from *PRSS1*^*Tg*^ mice, indicating that the inflammation increased. The microstructure changes of AP and CP tissues from *PRSS1*^*Tg*^ mice were not only reflected in mitochondrial swelling, karyopyknosis and ER disruption, but also the appearance of apoptotic bodies in AP tissues and mesenchyme in CP tissues (Fig. 2A). In comparison to the increase in MPO activity seen upon caerulein treatment in WT AP tissues, the increase in MPO activity in *PRSS1*^*Tg*^ AP tissues was significantly higher; this was confirmed by the MPO immunohistochemical score (Fig. S1A). Four weeks after caerulein injection, we observed a substantial decrease in the number of pancreatic parenchymal cells and an increase in fibrosis in pancreatic tissues from *PRSS1*^*Tg*^ CP mice (Fig. S1B). In comparison to caerulein-treated WT mice or untreated *PRSS1*^*Tg*^ mice, the expressions of Caspase-3 and PARP were significantly elevated in pancreatic tissues from *PRSS1*^*Tg*^ AP and CP mice, suggesting increased apoptosis (Fig. S1C). In addition, after caerulein treatment, the levels of the inflammatory cytokines IL-6, IL-1β, and TNF-α showed significantly higher in *PRSS1*^*Tg*^ mice than WT mice (Fig. S1D). These results demonstrate that in comparison to WT mice, *PRSS1*^*Tg*^ mice treated with caerulein could better mimic the development of human AP and CP, and can serve as a suitable model for mechanistic research on pancreatic inflammatory diseases.

### Proteomic analysis identifies EMC6 and APAF1 as potential regulatory molecules in AP and CP

To identify the regulatory proteins involved in pancreatitis development, proteomic analysis was performed on pancreatic tissues of *PRSS1*^*Tg*^ mice that were treated with caerulein or saline for 8 h and sacrificed 24 h later (Fig. S2A). As shown in the volcano plot, the red dot indicates significantly upregulated proteins, the green dot indicates significantly downregulated proteins, and the gray dot indicates proteins without significant change (Fig. 2B). These upregulated proteins and downregulated proteins were classified by protein Gene Ontology (GO) function (Fig. 2C). Using the selection criteria described in the “Materials and methods” section, a total of 499 DEPs were identified in this study. Then, we sorted proteins correlated with ER stress, cell death, and apoptosis in the Uniprot database among these 499 DEPs, 24 DEPs were identified (Fig. 2D), and a protein–PPI (protein–protein interaction) network of the 24 DEPs was constructed (Fig. 2E). The biological behavior of DEPs was classified by protein Kyoto Encyclopedia of Genes and Genomes (KEGG) function (Fig. S2B). To further identify proteins from among the 24 DEPs whose functions in ER stress strongly correlated with apoptosis, we searched the protein databases (Uniprot and String), and identified two proteins, EMC6 (Fig. 2F) and APAF1 (Fig. 2G). EMC6 is a newly ER-localized transmembrane protein that is involved in maintaining ER homeostasis^16^ and APAF1 is a key molecule in the intrinsic pathway of apoptosis^17^.

**Fig. 3:**
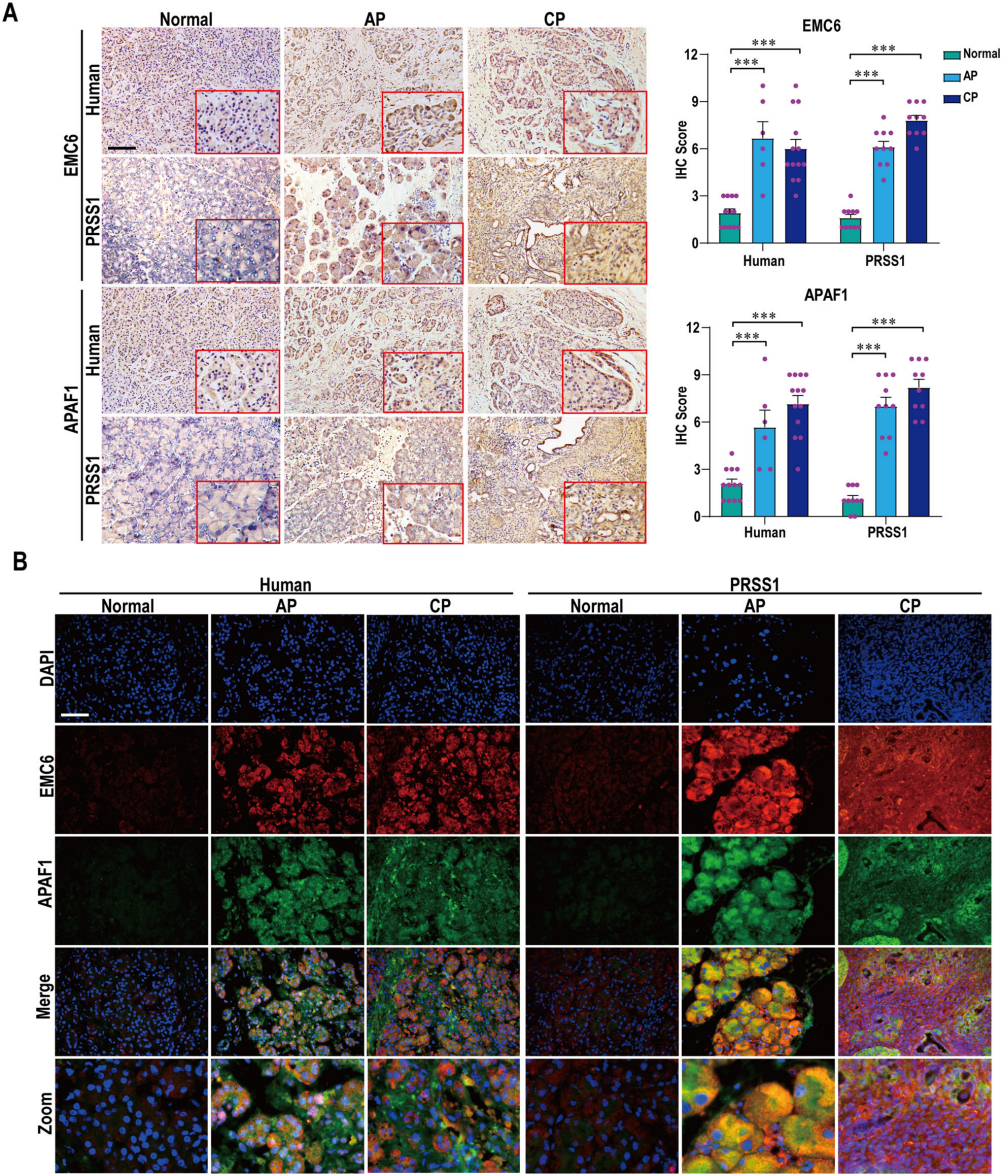
Elevated expressions of EMC6 and APAF1 in AP and CP tissues. **A** Representative images of immunohistochemistry staining of EMC6 and APAF1 in AP and CP tissues from human and *PRSS1*^*Tg*^ mice; immunohistochemistry scores were used to quantify the relative expressions of EMC6 and APAF1. **B** Immunofluorescence staining to examine the co-localization EMC6 (red fluorescence) and APAF1 (green fluorescence) in normal, AP and CP tissues from human and *PRSS1*^*Tg*^ mice. Data represents the mean ± SD; **P ≤ 0.01, ***P ≤ 0.001. Scale bars (immunohistochemistry) = 100 μm; scale bars (immunofluorescence) = 50 μm.

To validate above results, we examined the expressions of EMC6 and APAF1 in AP and CP tissues of patients and *PRSS1*^*Tg*^ mice by immunohistochemistry and immunofluorescence. Compared with healthy pancreatic tissues, the expressions of EMC6 and APAF1 were significantly increased in AP and CP tissues of humans and mice (Fig. 3A, B). Thus, we speculated that EMC6 and APAF1 probably correlated with pancreatic inflammatory diseases.

### APAF1 is regulated by EMC6, induces apoptosis to injure acinar cells and promotes inflammation

**Fig. 4:**
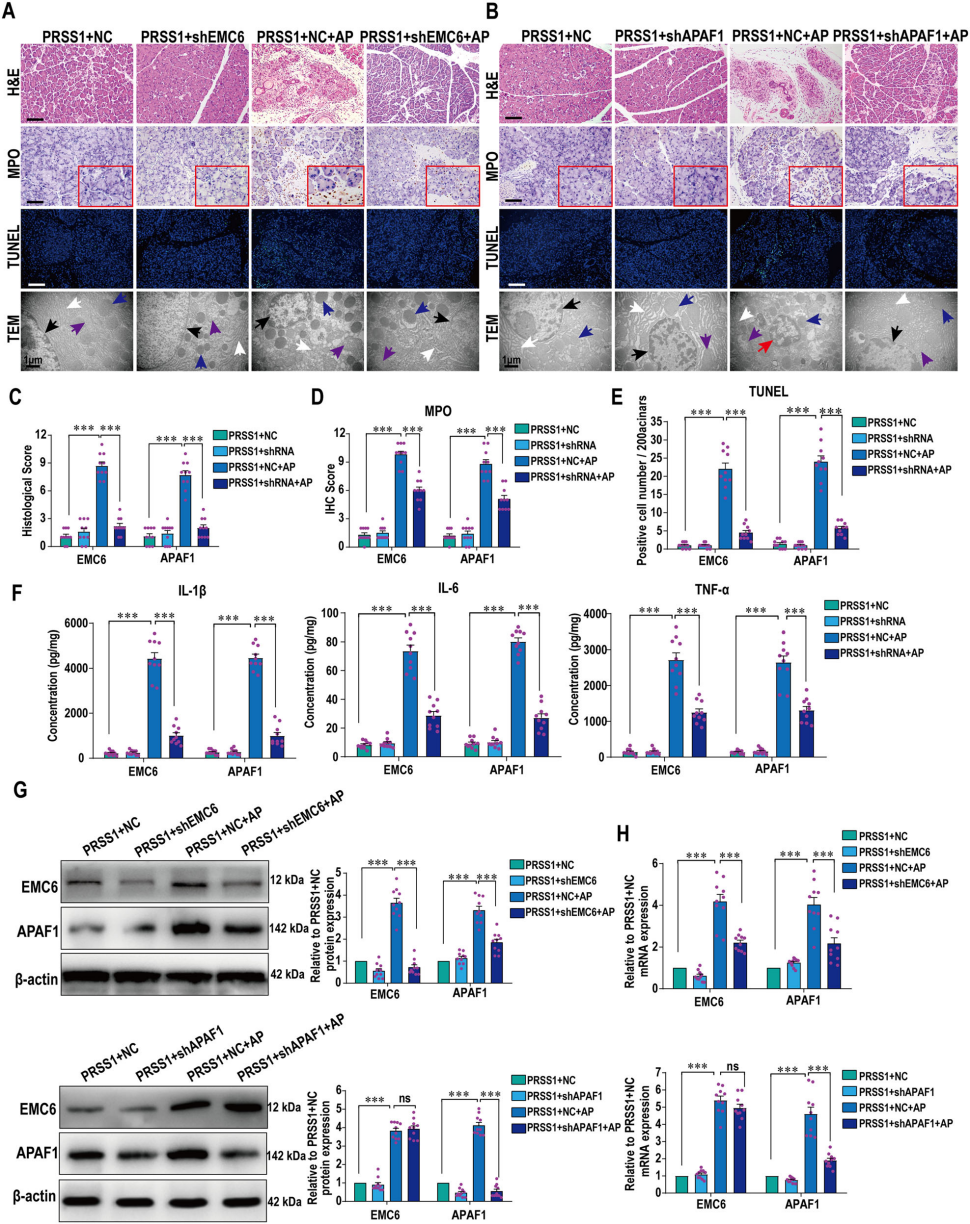
EMC6 and APAF1 induce apoptosis and promote AP progression. Histologic alteration, MPO expression, cell apoptosis, and microstructure changes in pancreatic tissues from *PRSS1*^*Tg*^ AP mice with EMC6 inhibition by shEMC6 (**A**) or APAF1 inhibition by shAPAF1 (**B**) were measured by H&E staining, IHC, transferase-mediated d-UTP nick-end-labeling (TUNEL) assays, and TEM, respectively; black arrows (↑): cell nucleus; white arrows (↑): endoplasmic reticulum; blue arrows (↑): zymogen granule; purple arrows (↑): mitochondria; red arrows (↑): apoptotic body. Histological scores (**C**), MPO immunohistochemistry scores (**D**), and TUNEL assays (**E**) of pancreatic tissues in *PRSS1*^*Tg*^ AP model after finishing EMC6 or APAF1 inhibition. **F** Expressions of IL-1β, IL-6, and TNF-α in pancreatic tissues from caerulein-treated *PRSS1*^*Tg*^ mice treated with shEMC6 or shAPAF1. **G** Western blotting analyses of EMC6 and APAF1 expressions in pancreatic tissues from *PRSS1*^*Tg*^ AP model; β-actin served as the internal loading control. **H** qRT-PCR for analysis of EMC6 and APAF1 expressions in pancreatic tissues from *PRSS1*^*Tg*^ AP model. NC, negative control. Data represents the mean ± SD; ns, no significant difference; *P ≤ 0.05, **P ≤ 0.01, ***P ≤ 0.001. Scale bars (H&E, MPO) = 100 μm; scale bars (TUNEL) = 200 μm.

**Fig. 5:**
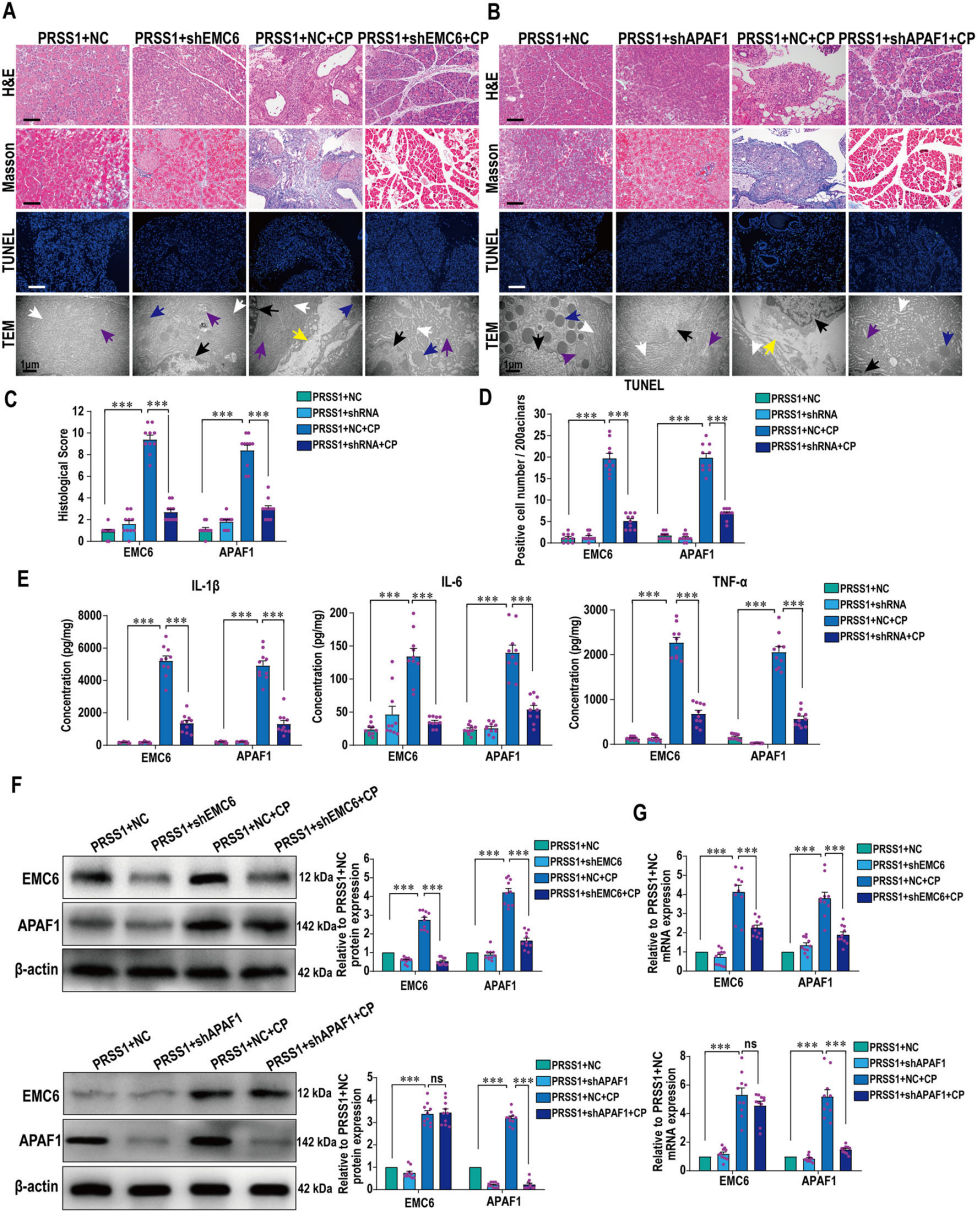
EMC6 and APAF1 induce apoptosis and promote CP progression. Histologic alteration, collagen deposition, cell apoptosis, and microstructure changes in pancreatic tissues from *PRSS1*^*Tg*^ CP model with EMC6 inhibition by shEMC6 (**A**) or APAF1 inhibition by shAPAF1 (**B**) were measured by H&E, Masson’s trichrome staining, TUNEL assays, and TEM, respectively; black arrows (↑): cell nucleus; white arrows (↑): endoplasmic reticulum; blue arrows (↑): zymogen granule; purple arrows (↑): mitochondria; yellow arrows (↑): mesenchyme. Histological scores (**C**) and TUNEL assays (**D**) of pancreatic tissues in *PRSS1*^*Tg*^ mice. **E** IL-6, IL-1β, and TNF-α pancreatic tissues concentrations in *PRSS1*^*Tg*^ mice with EMC6 or APAF1 inhibition after induction of CP. **F** Western blotting analyses of EMC6 and APAF1 expressions in pancreatic tissues from *PRSS1*^*Tg*^ CP model; β-actin served as the internal loading control. **G** qRT-PCR for analysis of EMC6 and APAF1 expressions in pancreatic tissues from *PRSS1*^*Tg*^ CP model. Data represents the mean ± SD; ns no significant difference; *P ≤ 0.05, **P ≤ 0.01, ***P ≤ 0.001. Scale bars (H&E, Masson) = 100 μm; scale bars (TUNEL) = 200 μm.

To verify that EMC6 and APAF1 play a role in pancreatitis, shRNA-mediated inhibition of their expressions through adenovirus-mediated transduction was performed in pancreatic tissues from *PRSS1*^*Tg*^ AP and CP mice, followed by assessment of cellular changes. The increase in cell deformation, MPO activity or collagen accumulation, number of apoptotic cells and ER damage seen in mouse pancreatic tissues upon AP or CP induction was greatly attenuated upon EMC6 or APAF1 inhibition (Figs. 4A–E and 5A–D). Consistent with the observations on apoptotic cell numbers (Figs. 4E and 5D), the increase in the expressions of Caspase-3 and PARP seen in mouse pancreatic tissues upon AP or CP induction was markedly attenuated upon the inhibition of EMC6 or APAF1 (Fig. S3A, B and S4A, B). Consistent with the histological findings on inflammatory cell infiltration (Figs. 4A, B and 5A, B), the increase in the levels of the inflammatory cytokines IL-6, IL-1β, and TNF-α in serum from the *PRSS1*^*Tg*^ AP mice (Fig. 4F) and in pancreatic tissues from *PRSS1*^*Tg*^ CP mice (Fig. 5E) was attenuated upon the inhibition of EMC6 or APAF1. The levels of serum amylase were significantly increased in *PRSS1*^*Tg*^ AP mice, but that were weaken after inhibition of EMC6 or APAF1 (Fig. S3C). Pancreatic edema was significantly induced in *PRSS1*^*Tg*^ AP mice and markedly decreased in *PRSS1*^*Tg*^ CP mice, which was negated upon inhibition of EMC6 or APAF1 (Figs. S3D and S4C).

**Fig. 6:**
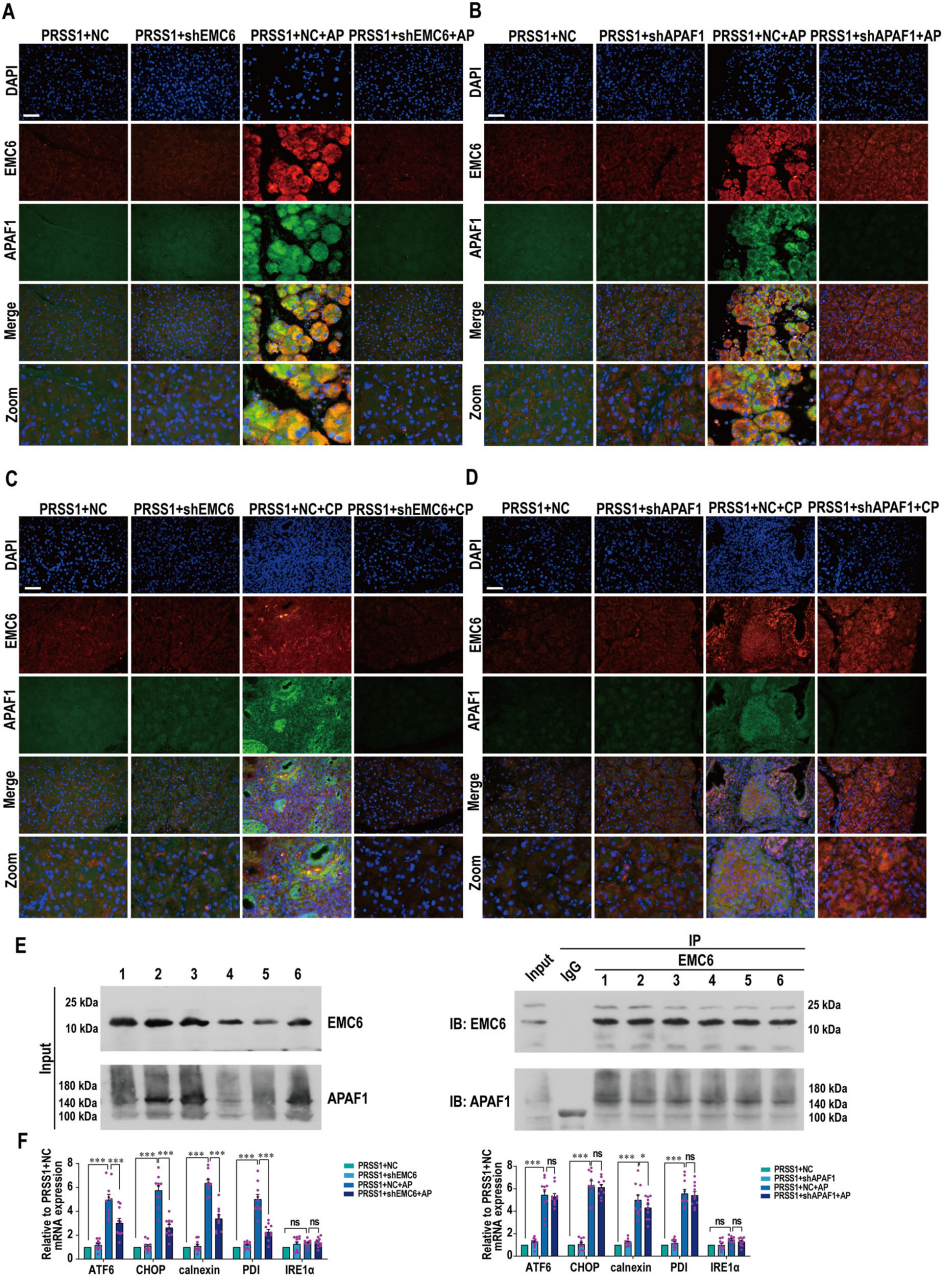
Expression of APAF1 is decreased upon EMC6 inhibition in *PRSS1*^*Tg*^ AP and CP model. Immunofluorescence staining to examine the co-localization of EMC6 (red fluorescence) and APAF1 (green fluorescence) in pancreatic tissues from *PRSS1*^*Tg*^ mice AP model with EMC6 inhibition (**A**) or APAF1 (**B**) inhibition. Immunofluorescence staining to examine the co-localization of EMC6 (red fluorescence) and APAF1 (green fluorescence) in pancreatic tissues from *PRSS1*^*Tg*^ mice CP model with EMC6 inhibition (**C**) or APAF1 inhibition (**D**). **E** Six AP samples from *PRSS1*^*Tg*^ mice were immunoprecipitated with anti-EMC6 antibody and immunoblotted with the indicated antibodies, which was performed to examine whether EMC6 interacted with APAF1 in pancreatitis tissues. **F** qRT-PCR for analysis of the expression levels of ER stress markers ATF6, CHOP, calnexin, PDI, and IRE1α in pancreatic tissues from PRSS1^Tg^ mice. Scale bars = 50 μm.

Interestingly, following EMC6 inhibition in AP tissues, the expression of APAF1 was decreased, while the expression of EMC6 did not change following APAF1 reduction (Fig. 4G, H). Similarly, the increase in EMC6 expression upon CP induction was not affected by APAF1 reduction (Fig. 5F, G). Consistent with these results, immunofluorescence analysis revealed that the increase in expressions of EMC6 and APAF1 upon AP (Fig. 6A, B) or CP (Fig. 6C, D) induction, was attenuated upon inhibition of EMC6. However, upon inhibition of APAF1, the increase in active foci of EMC6 remained unchanged, while the increase in active foci of APAF1 was markedly attenuated (Fig. 6A–D). Furthermore, we validated whether EMC6 is associated with APAF1 by co-immunoprecipitation (Co-IP), results of which showed that EMC6 interacted with APAF1 during AP (Fig. 6E). In order to check the change of ER stress in the AP model in response to either EMC6 or APAF1 loss, we examined the expression of ER stress markers, such as ATF6, CHOP, calnexin, PDI, and IRE1α, by qRT-PCR. The expressions of ATF6, CHOP, calnexin, and PDI were significantly ameliorated upon EMC6 silencing in AP tissues, while the expression of IRE1α did not change. However, except calnexin, there were no significant changes in the expressions of other ER stress markers (including ATF6, CHOP, PDI, and IRE1α) following APAF1 knockdown.

Collectively, these results indicate that EMC6 and APAF1 promote apoptosis and inflammatory injury of acinar cells in pancreatic inflammatory diseases. Our results further suggest that APAF1 is regulated by EMC6, induces apoptosis to injure acinar cells, and promotes inflammation in the development of pancreatitis.

## Discussion

Until now, poor outcomes of AP and CP are still lying ahead of physicians^18^, due to ineffective treatment strategies from limited understanding of their pathogenesis. Therefore, understanding the regulatory mechanism of pancreatitis is essential for improving disease outcome. By proteome screening in *PRSS1*^*Tg*^ mice pancreatitis tissues, we identified EMC6 and APAF1 as being closely associated with ER stress and apoptosis seen in pancreatic inflammatory diseases. Moreover, we confirmed, for the first time, that activation of EMC6 could upregulate APAF1 expression, accompanied by elevated apoptosis and inflammation. Correspondingly, the inhibition of EMC6 could downregulate APAF1, while apoptosis and inflammation were attenuated.

Misfolded proteins can aggregate excessively in the ER in response to external stimuli, resulting in ER stress and leading to the induction of apoptosis if ER stress persists^19,20^. Meanwhile, the unfolded protein response (UPR) attempts to self-protect cells by translational attenuation to limit further protein accumulation, transcriptional activation of genes encoding ER-resident chaperones to increase the ER folding capacity, and induction of ER-associated degradation to remove misfolded molecules. If these mechanisms are not sufficient, stress-damaged cells are eliminated by a thorough induction of apoptosis^11^. ER stress responses being prevalent in AP, pancreatic acinar cells are particularly susceptible to ER perturbations and resulting cellular damage^11^. Recently, we demonstrated that ATF6, one of ER stress responsive proteins, promoted acinar apoptosis as CP progresses^14^.

The EMC family, which was first discovered in yeast, consists of six subunits (EMC1–6) in yeast and ten subunits (EMC1–10) in mammals^21,22^. EMC6, a newly identified ER-localized transmembrane protein, regulates autophagosome formation, and its deficiency causes an accumulation of autophagosome precursor structures and impairs autophagy^23^. Shen et al. revealed that EMC6 induces autophagy via the inactivation of PIK3CA/AKT/mTOR signaling and PIK3C3 activity^24^. The disruption of EMC affects a series of processes, including ER stress, protein trafficking, organelle communication, viral maturation, and lipid homeostasis^25^. Meanwhile, EMC6 has also been shown to induce cell death in gastric cancer cells^26^. Therefore, we speculated in the present study that EMC6 also induced apoptosis in the pathological context of pancreatitis, and found that ER stress responses and apoptosis were enhanced during pancreatitis progression. In this study, EMC6 was highly expressed and regulated the ER stress pathway, which influenced the outcomes of AP and CP. However, the mechanism by which EMC6 plays a regulatory role in pancreatitis needs to be further clarified.

The proteomic analysis also identified the apoptosis-related protein APAF1 as a potential regulatory molecule. APAF1 is the central component of the apoptosome, a multiprotein complex, that activates procaspase-9 after cytochrome C release from the mitochondria in the intrinsic pathway of apoptosis^27^. APAF1 is a principal regulator not only of cell death but also of cell recovery pathways. The inhibition of APAF1 allows cells to survive after activation of apoptosis and permits cell recovery after hypoxia or DNA damage^27^. In our study, APAF1 was highly expressed in the AP and CP tissues of patients and *PRSS1*^*Tg*^ mice. Furthermore, inflammation and acinar cell apoptosis increased with high APAF1 expression. The activation of EMC6 and APAF1 was upregulated, apoptosis was activated, and inflammation was aggravated. Moreover, we obtained the opposite results after the inhibition of EMC6. Our study also showed that following EMC6 inhibition in pancreatitis tissues, the expressions of ER stress markers and APAF1 were ameliorated. Except calnexin, but there were no significant changes in other ER stress markers (including EMC6) following APAF1 silencing. Based on the above findings, we can conclude that APAF1 is regulated by EMC6 and that APAF1-induced apoptosis leads to pancreatic acinar cell injury and subsequent pancreatitis.

The inactivation of EMC6 causes embryonic lethality or developmental arrest and decrease the expression of levamisole-type acetylcholine receptors (L-AChRs)^16^. Moreover, mutations in EMC6 lead to rhodopsin homeostasis defects, which may cause retinal degeneration^28^. Furthermore, EMC6 overexpression significantly inhibited cancer cell growth, induced apoptosis, suppressed the migration and invasion, and exerted strong antitumor activity in gastric cancer cells^26,29^. APAF1 is a key molecule in the intrinsic pathway of apoptosis, and apoptosis dysregulation is at the root of a variety of diseases. Some pathological conditions, such as stroke, neurodegenerative diseases and cancer, are the result of dysregulation of the intrinsic apoptosis pathway^17^. Previous research found that siAPAF1 significantly promoted pancreatic cancer cell proliferation and repressed apoptosis; expression of exogenous APAF1 could rescue these defects^30^. APAF1-deficient mice showed severe brain malformations due to apoptosis deficiency, which might be partially ascribed to alterations in the centrosome and Golgi and axonal elongation caused by APAF1 deletion^31^. Furthermore, the formation of mutant huntingtin-dependent aggregates and cell death would be reduced upon the inactivation of APAF1^32^.

In conclusion, by using suitable AP and CP models, we have defined the regulatory relationship between EMC6 and APAF1: EMC6 regulates acinar cell injury via APAF1 in pancreatic inflammatory diseases. These findings may help us to increase our understanding on the ER stress response and apoptosis molecular pathways. However, the exact mechanism by which EMC6 and/or APAF1 regulate the progression of pancreatitis is unclear and deserves further investigation.

## Access to data

The mass spectrometry proteomics data have been deposited to the ProteomeXchange Consortium via the PRIDE partner repository with the dataset identifier PXD016651.

## Supplementary information

Supplementary Figure and Table Legends

Figure S1

Figure S2

Figure S3

Figure S4

Supplemental Methods

Table S1. Clinical characteristic data of patients and normal controls

Table S2. The sequences of the primers used
