# Supplementary material for: EMC6 regulates acinar apoptosis via APAF1 in acute and chronic pancreatitis
Source: Cell Death Dis. 2020 Nov 11;11(11):966. doi: 10.1038/s41419-020-03177-3 (PMC7658364; doi:10.1038/s41419-020-03177-3)
Supplement: Supplementary file 1 — Supplementary Figure and Table Legends [file 41419_2020_3177_MOESM1_ESM.docx]

**Supplementary Figure and Table Legends**

**Figure S1. *PRSS1^Tg^* mice could better mimic the development of human AP and CP.**

**(A)** The MPO immunohistochemistry and immunohistochemistry scores of AP tissues from WT and *PRSS1^Tg^* mice. (**B)** Analysis of collagen deposition in CP tissues from WT and *PRSS1^Tg^* mice by Masson’s trichrome staining. **(C)** Pancreas sections (including normal, AP, CP) from WT and *PRSS1^Tg^* mice were stained for apoptosis-related Caspase-3 and PARP. **(D)** The levels of IL-1β, IL-6, and TNF-α in pancreatic tissues from WT and *PRSS1^Tg^* mice treated with caerulein for AP and CP induction. Cer, caerulein. Data represents the mean ± SD; ns, no significant difference; * *P* ≤ 0.05, ** *P* ≤ 0.01, *** *P* ≤ 0.001. Scale bars = 100 μm.

**Figure S2. Proteomics analysis scheme and the Kyoto Encyclopedia of Genes and Genomes (KEGG) functional classification.**

**(A)** Schematic representation of protocol followed for proteomic analysis. *PRSS1^Tg^* mice were treated with caerulein or saline for 8 hours and sacrificed 24 hours later, the pancreatic tissues were harvested for proteomic analysis. **(B)** Pathway annotation histogram, the X-axis represents the number of protein annotations and the Y-axis represents the KEGG functional classification; the KEGG metabolic pathway contains six branches: Cellular Processes, Environmental Information Processing, Genetic Information Processing, Human Diseases (animals only), Metabolism, Organic Systems.

**Figure S3. Inhibition of EMC6 or APAF1 expression** **ameliorates apoptosis and AP progression.**

Immunohistochemistry and scores for apoptosis-related Caspase-3 and PARP in pancreatic tissues from *PRSS1^Tg^* AP model with EMC6 **(A)** or APAF1 **(B)** inhibition. Serum amylase activity **(C)** and degree of edema **(D)** in pancreatic tissues from *PRSS1^Tg^* AP model with EMC6 or APAF1 inhibition. **(E)** Schematic diagrams of shEMC6, NC-EMC6, shAPAF1, and NC-APAF1. **(F)** The transfection efficacy of adenovirus (including shEMC6 and shAPAF1) by quantification of green fluorescent protein (GFP) or red fluorescent protein (RFP)-positive acinars were assessed by fluorescence microscope. Data represents the mean ± SD; * *P* ≤ 0.05, ** *P* ≤ 0.01. Scale bars (immunohistochemistry) = 100 μm; scale bars (immunofluorescence) = 50 μm.

**Figure S4**. **Inhibition of EMC6 or APAF1 expression ameliorates apoptosis and CP progression.**

Representative images of immunohistochemistry staining of apoptosis-related Caspase-3 and PARP in pancreatic tissues from *PRSS1^Tg^* mice CP model with EMC6 **(A)** or APAF1 **(B)** inhibition. **(C)** Degree of edema in pancreatic tissues from *PRSS1^Tg^* CP model with EMC6 or APAF1 inhibition. Data represents the mean ± SD; ** *P* ≤ 0.01. Scale bars = 100 μm.

**Table S1.** Clinical characteristic data of patients and normal controls.

**Table S2.** The sequences of the primers used.
