# Supplementary figures and images for: EMC6 regulates acinar apoptosis via APAF1 in acute and chronic pancreatitis
Source: Cell Death Dis. 2020 Nov 11;11(11):966. doi: 10.1038/s41419-020-03177-3 (PMC7658364; doi:10.1038/s41419-020-03177-3)

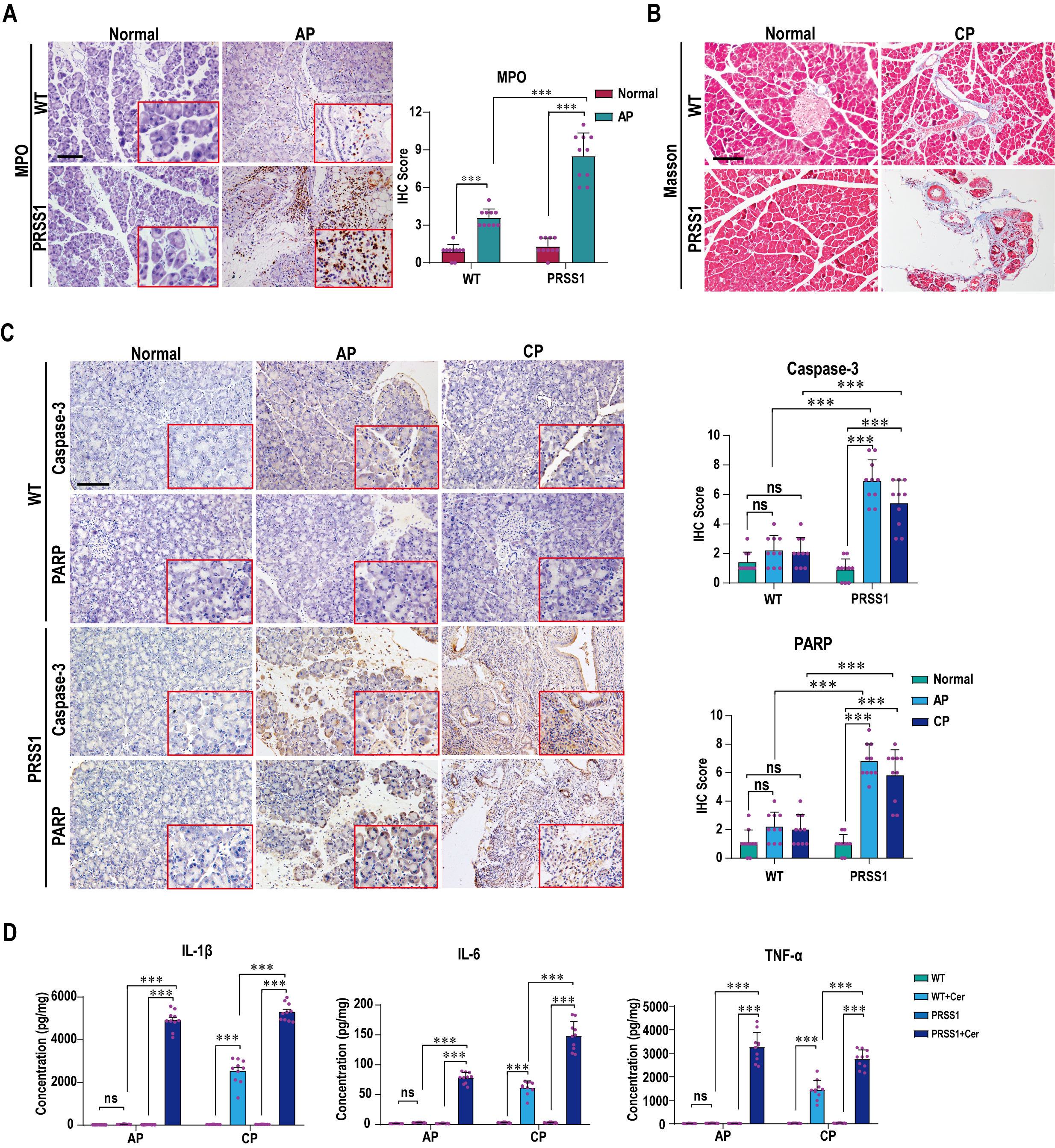

Supplement: Supplementary file 2 — Figure S1 [file 41419_2020_3177_MOESM2_ESM.tif]

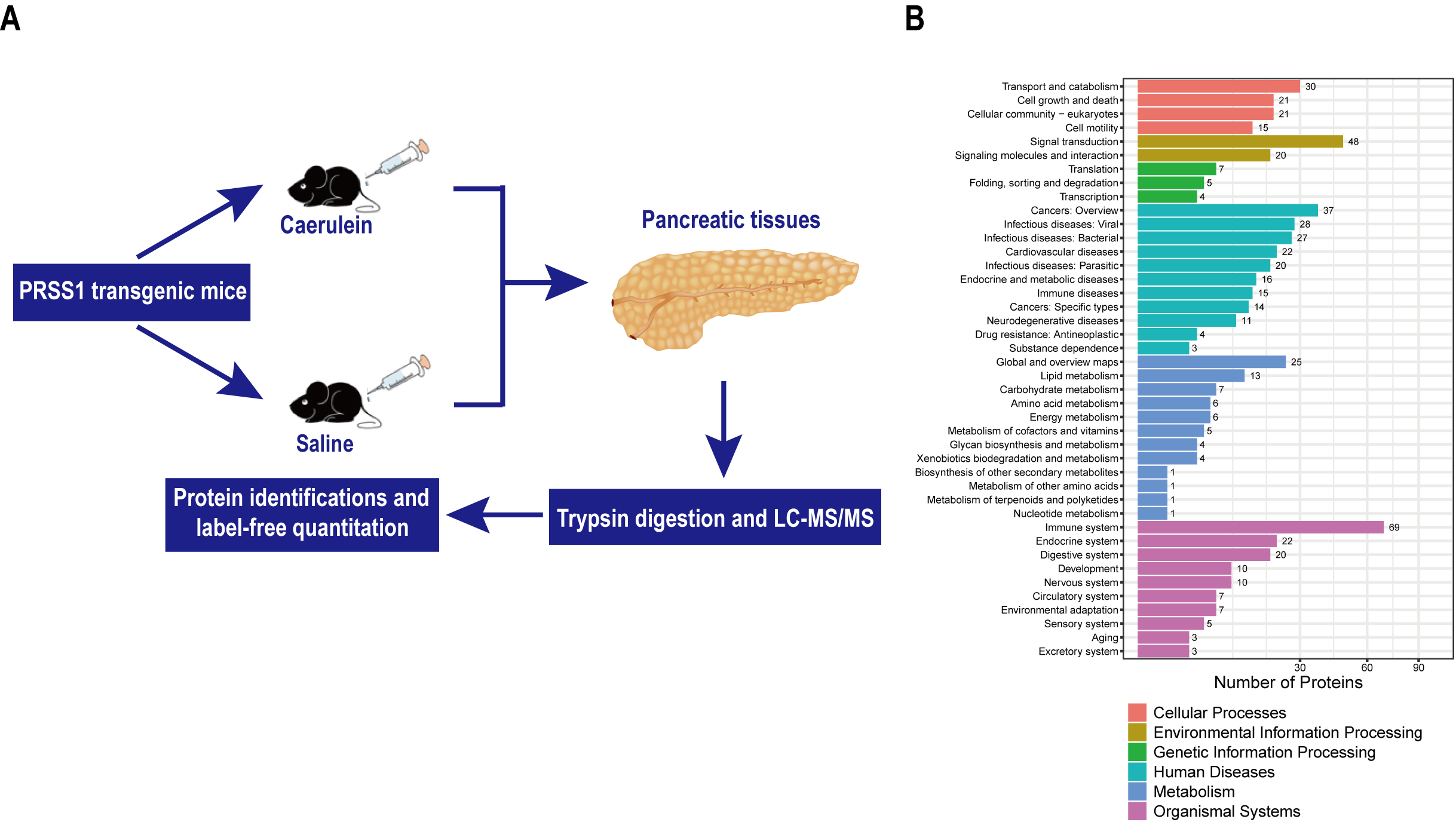

Supplement: Supplementary file 3 — Figure S2 [file 41419_2020_3177_MOESM3_ESM.tif]

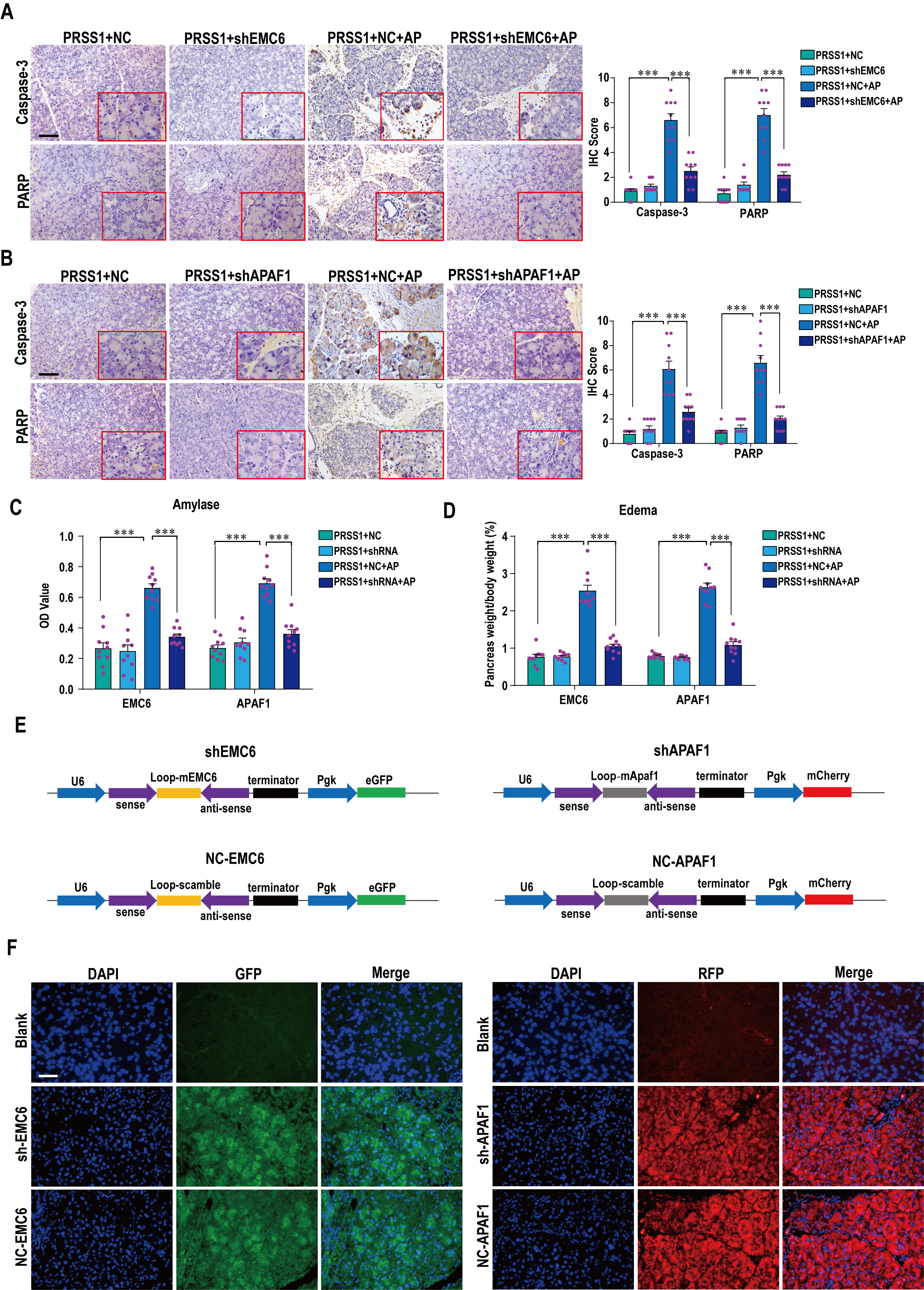

Supplement: Supplementary file 4 — Figure S3 [file 41419_2020_3177_MOESM4_ESM.tif]

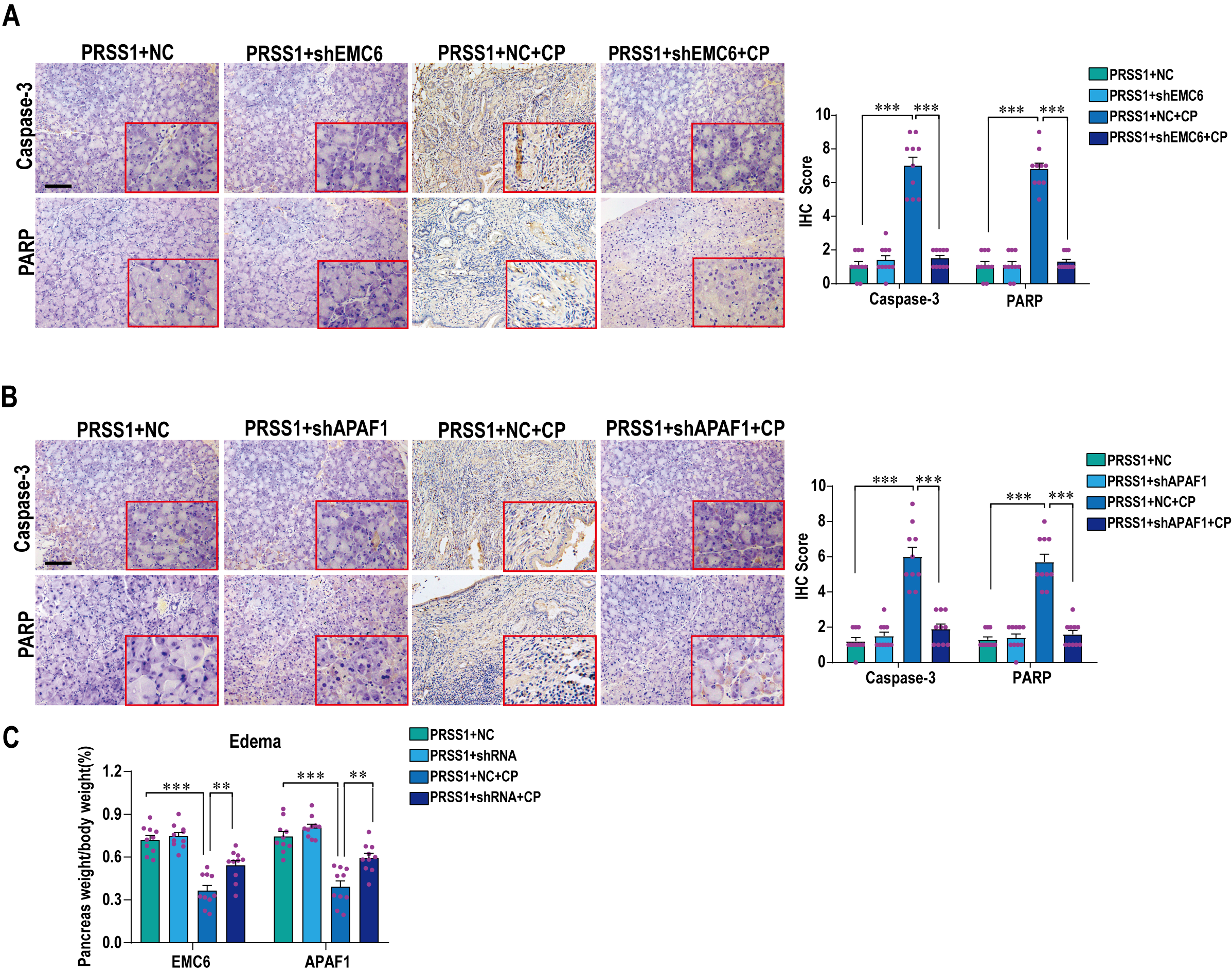

Supplement: Supplementary file 5 — Figure S4 [file 41419_2020_3177_MOESM5_ESM.tif]
