# Supplementary material for: EMC6 regulates acinar apoptosis via APAF1 in acute and chronic pancreatitis
Source: Cell Death Dis. 2020 Nov 11;11(11):966. doi: 10.1038/s41419-020-03177-3 (PMC7658364; doi:10.1038/s41419-020-03177-3)
Supplement: Supplementary file 6 — Supplemental Methods [file 41419_2020_3177_MOESM6_ESM.docx]

**Supplementary Methods**

***PRSS1 transgenic (PRSS1^Tg^) mice, adenoviral shRNA for EMC6 and APAF1 knockdown***

The sex and age matched mice were sacrificed for further assays. Single transgenic littermates subjected to the same treatment were used as controls. All mouse colonies were maintained in a specific pathogen-free barrier facility at the Nanfang Hospital, Southern Medical University laboratory animal center.

To investigate the effect of EMC6 on apoptosis and inflammation, pancreatic tissues from *PRSS1^Tg^* mice were infected with adenovirus vectors harboring a shEMC6 fragment for EMC6 inhibition or scramble shRNA for negative control. Similarly, pancreatic tissues from *PRSS1^Tg^* mice were infected with adenovirus vectors harboring a shAPAF1 fragment for APAF1 inhibition or scramble shRNA for negative control (NC) (Figure S3E). After adenoviral transfection (including shEMC6, NC-EMC6, shAPAF1, and NC-APAF1) for 48 h, we assessed the transfection efficacy of adenovirus by quantification of green or red fluorescent protein (GFP or RFP)-positive cells under a fluorescence microscope. Microscopic examination revealed that the majority of pancreatic acinar cells were strongly GFP or RFP positive (Figure S3F). All adenovirus vectors were purchased from Vector Builder Inc. (Guangzhou, China). The sequences of the primers used are listed in Supplementary Table S2.

The EMC6/APAF1-deficient *PRSS1^Tg^* mice were injected with adenovirus three days prior to caerulein injection. Virus suspension with a titer of 1×10^12^ was injected into the pancreatic tissue at a dose of 10-15 μl/mouse (mass 20-25 g). Five points of pancreatic tissue was selected for adenovirus injection each time. After the virus injection, caerulein injection was then performed subsequently.

***Immunohistochemistry***

Immunohistochemistry assays were carried out on human pancreatic samples and mice pancreatic tissues to detect and score EMC6, APAF1, collagen I, MPO, Caspase-3, and PARP expression. Pancreatic tissues collected from patients and mice were fixed in 4% neutral phosphate-buffered formalin at 4 °C. Hematoxylin and eosin (H&E) staining was performed using 5-mm samples of pancreas by experienced pathologists at the Pathological Department of Southern Medical University and histological evaluation was performed in a double-blinded manner. The immunohistochemistry assay based on the horseradish peroxidase system was performed using specific antibodies targeting EMC6 (Bioss; diluted 1: 100), APAF1 (Proteintech; diluted 1: 50), collagen I (Thermo Fisher; diluted 1:500), MPO (Abcam; diluted 1: 25), Caspase-3 (GeneTex; diluted 1:100), PARP (GeneTex; diluted 1:100). Positive staining of cells was observed and separately evaluated by two experienced pathologists.

***Immunofluorescence assay***

For immunofluorescence and visualizing EMC6, APAF1 and α-SMA expression in pancreatic tissues, tissue slides were first incubated with antibodies against EMC6 (Bioss; diluted 1: 100), APAF1 (Proteintech; diluted 1: 50), α-SMA (Abcam; diluted 1:500), at room temperature for 1 h followed by incubation with fluorescein isothiocyanate or phycoerythrin-conjugated secondary antibodies (Sangon, Shanghai, China). Pancreatic tissues were also stained with DAPI. Tissue slides were observed using a fluorescent microscope.

***Western blotting and qRT-PCR***

Using the Tissue Total Protein Extraction Kit (C510003; Sangon, Shanghai, China) to extract total protein from the mice pancreatic tissues following the manufacturer’s instructions. After protein concentration determination, approximately 25 μg protein from each sample was denatured at 100°C for 5 min, separated by 12% SDS-PAGE, and transferred onto a PVDF membrane (Millipore). After overnight blocking with 5% skim milk, membranes were incubated overnight with primary antibodies: anti-EMC6 (diluted 1:500; Bioss), anti-APAF1 (diluted 1:500; Proteintech), and anti-β-actin (diluted 1:500; Abcam) at 4 °C, washed three times with PBS solution for 5 min, and incubated with horseradish peroxidase-conjugated secondary antibodies for 2 h. The immunocomplexes were finally developed with enhanced chemiluminescence solution (Thermo Fisher Scientific). β-actin was used as the internal standard.

The mRNA expression levels of EMC6 and APAF1 in this study were determined by Quantitative RT-PCR. Total RNA samples from pancreatic tissues were extracted using the TRIzol reagent (Thermo Fisher Scientific) as instructed by the manufacturer. Approximately 3.0 μg total RNA samples from each group were collected for cDNA synthesis followed by PCR analysis. The sequences of the primers used are listed in Supplementary Table S2. The protein abundance and Gene mRNA levels were evaluated with at least three biological replicates.

***Transferase-mediated d-UTP nick-end-labeling (TUNEL) assay***

To detect apoptosis, Terminal deoxynucleotidyl TUNEL was performed. Pancreatic tissues were embedded in routine paraffin, de-waxed using dimethyl benzene, and hydrated using gradient ethanol. Apoptosis was determined by using a TdT Frag DNA Fragmentation Imaging kit (Sigma Aldrich) according to the manufacturer’s instructions. Green staining in the nuclei of positive cells containing labeled DNA fragments indicated internucleosomal DNA cleavage. Three representative slides were chosen and five random visions of high field (× 200) were analyzed.
