# Supplementary material for: EMC6 regulates acinar apoptosis via APAF1 in acute and chronic pancreatitis
Source: Cell Death Dis. 2020 Nov 11;11(11):966. doi: 10.1038/s41419-020-03177-3 (PMC7658364; doi:10.1038/s41419-020-03177-3)
Supplement: Supplementary file 7 — Table S1. Clinical characteristic data of patients and normal controls [file 41419_2020_3177_MOESM7_ESM.doc]

**Table S1. Clinical characteristic data of patients and normal controls**

| **NO.** | **age** | **Gender** | **Disease** | **Group** |
| --- | --- | --- | --- | --- |
| 1 | 44 | female | Peritumoral | Normal |
| 2 | 48 | male | Peritumoral | Normal |
| 3 | 27 | male | Pancreas benign tumer | Normal |
| 4 | 43 | female | Peritumoral | Normal |
| 5 | 70 | male | Peritumoral | Normal |
| 6 | 69 | female | Peritumoral | Normal |
| 7 | 43 | male | Pancreas benign tumer | Normal |
| 8 | 56 | female | Peritumoral | Normal |
| 9 | 34 | male | Pancreas benign tumer | Normal |
| 10 | 42 | female | Peritumoral | Normal |
| 11 | 49 | male | Peritumoral | Normal |
| 12 | 37 | female | Pancreas benign tumer | Normal |
| 13 | 42 | male | Pancreatic pseudocyst | AP |
| 14 | 53 | male | Acute biliary pancreatitis | AP |
| 15 | 44 | female | Acute necrotic pancreatitis | AP |
| 16 | 49 | female | Acute necrotic pancreatitis | AP |
| 17 | 55 | male | Acute biliary pancreatitis | AP |
| 18 | 47 | male | Pancreatic pseudocyst | AP |
| 19 | 42 | female | Chronic pancreatitis | CP |
| 20 | 20 | male | Chronic pancreatitis | CP |
| 21 | 39 | male | Chronic pancreatitis | CP |
| 22 | 34 | male | Chronic pancreatitis | CP |
| 23 | 57 | male | Chronic pancreatitis | CP |
| 24 | 75 | female | Chronic pancreatitis | CP |
| 25 | 31 | male | Chronic pancreatitis | CP |
| 26 | 43 | male | Chronic pancreatitis | CP |
| 27 | 42 | male | Chronic pancreatitis | CP |
| 28 | 38 | male | Chronic pancreatitis | CP |
| 29 | 42 | male | Chronic pancreatitis | CP |
| 30 | 78 | female | Chronic pancreatitis | CP |
| 31 | 69 | male | Chronic pancreatitis | CP |
