# Supplementary material for: EMC6 regulates acinar apoptosis via APAF1 in acute and chronic pancreatitis
Source: Cell Death Dis. 2020 Nov 11;11(11):966. doi: 10.1038/s41419-020-03177-3 (PMC7658364; doi:10.1038/s41419-020-03177-3)
Supplement: Supplementary file 8 — Table S2. The sequences of the primers used [file 41419_2020_3177_MOESM8_ESM.doc]

**Table S2. The sequences of the primers used**

| **Primer ID** | **Sequences (5’-3’)** |
| --- | --- |
| **PRSS1** | (F) GAGCGGATTTGAACGTTGTG |
|  | (R) TACTTGAAGAGATTTGGCGG |
| **EMC6** | (F) GTCGCCAAGATTTGCTCCCT |
|  | (R) AAACACACAATGCCGGTACAC |
| **APAF1** | (F) GATCCACACAGGCCATCACA |
|  | (R) GGCGGGAGTCTATGTTCCAC |
| **ATF6** | (F) CGCCTTTTAGTCCGGTTCTT |
|  | (R) CCAGTTGGTAACAATGCCATGT |
| **CHOP** | (F) ACCTTCACTACTCTTGACCCTG |
|  | (R) GATGTGCGTGTGACCTCTGT |
| **IRE1α** | (F) GGATGCCTGGTTTGTAGTGGA  5’-TGAGTAGCGGCGATAGGTTGT-3’(R |
|  | (R) TGAGTAGCGGCGATAGGTTGT |
| **Calnexin** | (F) GCATCATGCCATCTCTGCT |
|  | (R) AGCAGCTTCACATAGGCACC |
| **PDI** | (F) ACTGCAGCTGCAGAGTCCTT |
|  | (R) TGGTACTTGGAGAACACACCAC |
| **β-actin** | (F) GAGGTATCCTGACCCTGAAGTA |
|  | (R) CACACGCAGCTCATTGTAGA |
